# Effects of DNA, RNA, and Protein Methylation on the Regulation of Ferroptosis

**DOI:** 10.7150/ijbs.85454

**Published:** 2023-07-09

**Authors:** Xiancan Wang, Xianghai Kong, Xin Feng, Ding-Sheng Jiang

**Affiliations:** 1 Department of Cardiovascular Surgery, The Central Hospital of Wuhan, Tongji Medical College, Huazhong University of Science and Technology, Wuhan, 430014, Hubei, China.; 2 Department of Intervention & Vascular Surgery, The Central Hospital of Wuhan, Tongji Medical College, Huazhong University of Science and echnology, Wuhan, 430014, Hubei, China.; 3 Division of Cardiovascular Surgery, Tongji Hospital, Tongji Medical College, Huazhong University of Science and Technology, Wuhan, Hubei, China.; 4 Key Laboratory of Organ Transplantation, Ministry of Education; NHC Key Laboratory of Organ Transplantation; Key Laboratory of Organ Transplantation, Chinese Academy of Medical Sciences, Wuhan, Hubei, China.

**Keywords:** Ferroptosis, DNA methylation, RNA m^6^A methylation, Protein methylation, Histone methylation, Disulfidptosis

## Abstract

Ferroptosis is a form of programmed cell death characterized by elevated intracellular ferrous ion levels and increased lipid peroxidation. Since its discovery and characterization in 2012, considerable progress has been made in understanding the regulatory mechanisms and pathophysiological functions of ferroptosis. Recent findings suggest that numerous organ injuries (*e.g.,* ischemia/reperfusion injury) and degenerative pathologies (*e.g.,* aortic dissection and neurodegenerative disease) are driven by ferroptosis. Conversely, insufficient ferroptosis has been linked to tumorigenesis. Furthermore, a recent study revealed the effect of ferroptosis on hematopoietic stem cells under physiological conditions. The regulatory mechanisms of ferroptosis identified to date include mainly iron metabolism, such as iron transport and ferritinophagy, and redox systems, such as glutathione peroxidase 4 (GPX4)-glutathione (GSH), ferroptosis-suppressor-protein 1 (FSP1)-CoQ_10_, FSP1-vitamin K (VK), dihydroorotate dehydrogenase (DHODH)-CoQ, and GTP cyclohydrolase 1 (GCH1)-tetrahydrobiopterin (BH_4_). Recently, an increasing number of studies have demonstrated the important regulatory role played by epigenetic mechanisms, especially DNA, RNA, and protein methylation, in ferroptosis. In this review, we provide a critical analysis of the molecular mechanisms and regulatory networks of ferroptosis identified to date, with a focus on the regulatory role of DNA, RNA, and protein methylation. Furthermore, we discuss some debated findings and unanswered questions that should be the foci of future research in this field.

## Introduction

**Figure 1:**
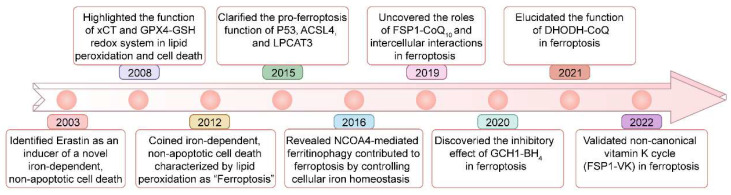
** Key milestones in the study of ferroptosis over time**.

Ferroptosis is regulated cell death driven by iron overload-triggered lipid peroxidation and is involved in a wide range of diseases, including cancers, cardiovascular diseases, degenerative diseases, infectious diseases, autoimmune diseases, and ischemia/reperfusion injury^1^-^7^. Although first defined in 2012, as early as 2003, when high-throughput screening revealed compounds with the ability to kill engineered tumorigenic cells, this nonapoptotic cell death, now known as ferroptosis, was initially characterized and shown to be induced by Erastin^1^, ^8^, ^9^ **(Figure 1)**. Five years later (in 2008), the importance of ferrous iron, the system xc^-^ (xCT) controlled cystine/cysteine redox cycle, and the glutathione peroxidase 4 (GPX4)-glutathione (GSH) redox system in lipid peroxidation and non-apoptotic cell death was highlighted by different research groups^10^-^12^. In 2012, ferrostatin-1, the first inhibitor of ferroptosis, was discovered by Brent R Stockwell and his colleagues^1^. Since then, considerable advances have been made in understanding the pathological functions of ferroptosis, such as its roles in tumorigenesis and degenerative diseases^13^-^15^, but its precise physiological function remained unknown. Recently, Zhao *et al.* demonstrated that histone deubiquitinase MYSM1 (Myb-like, SWIRM and MPN domains 1) deficiency reduced the translation of ferroptosis-protective mRNAs, resulting in increased ferroptosis of human hematopoietic stem cells (HSCs), and HSC population maintenance was fully restored by the ferroptosis inhibitors (*e.g.,* deferoxamine (DFO), ferrostatin-1 (Fer-1), and vitamin E)^16^. This study revealed the specific vulnerability of HSCs to ferroptosis and a unique physiological role played by ferroptosis in human hematopoiesis.

In recent years, many key molecular mechanisms of ferroptosis have been identified, including GPX4-GSH, ferroptosis-suppressor-protein 1 (FSP1)-CoQ_10_, GTP cyclohydrolase 1 (GCH1)-tetrahydrobiopterin (BH_4_), dihydroorotate dehydrogenase (DHODH)-CoQ, and FSP1-vitamin K (VK)^8^, ^17^-^19^ **(Figure 1)**. Additionally, epigenetic mechanisms such as DNA methylation, RNA methylation, protein methylation, and noncoding RNAs have gained increased attention. For instance, DNA methylation of key ferroptosis-related genes, such as GPX4, FSP1 and NFE2-like BZIP transcription factor 2 (NRF2) genes, has been shown to affect the expression of these genes, which participate in the regulation of ferroptosis^20^-^22^. Furthermore, prediction models combining RNA m^6^A modification regulators with ferroptosis-related genes have been established to effectively reflect the progression and prognosis of tumors^23^, and the RNA m^6^A modification can also regulate the fate of ferroptosis-related mRNAs, thereby controlling their protein expression^3^, ^24^. Targeting protein methylation factors has been shown to improve the prognosis of related diseases by regulating ferroptosis. For example, we recently found that the histone methyltransferase inhibitor BRD4770 can delay the pathological progression of aortic dissection by inhibiting ferroptosis^4^. There have been major advances in our understanding of the mechanisms related to DNA, RNA, and protein methylation that govern ferroptosis. Therefore, it is necessary to summarize and review the research progress in this field.

Many reviews have summarized and discussed in detail the regulatory mechanisms, biology, and roles of ferroptosis in disease^2^, ^8^, ^13^, ^25^-^29^, and readers interested in a more in-depth understanding can consult the relevant literature. In the present review, we focus on the effects of DNA methylation, the RNA m^6^A modification, and protein methylation and the underlying mechanisms of these effects on ferroptosis.

## An overview of ferroptosis

### Lipid peroxidation

**Figure 2:**
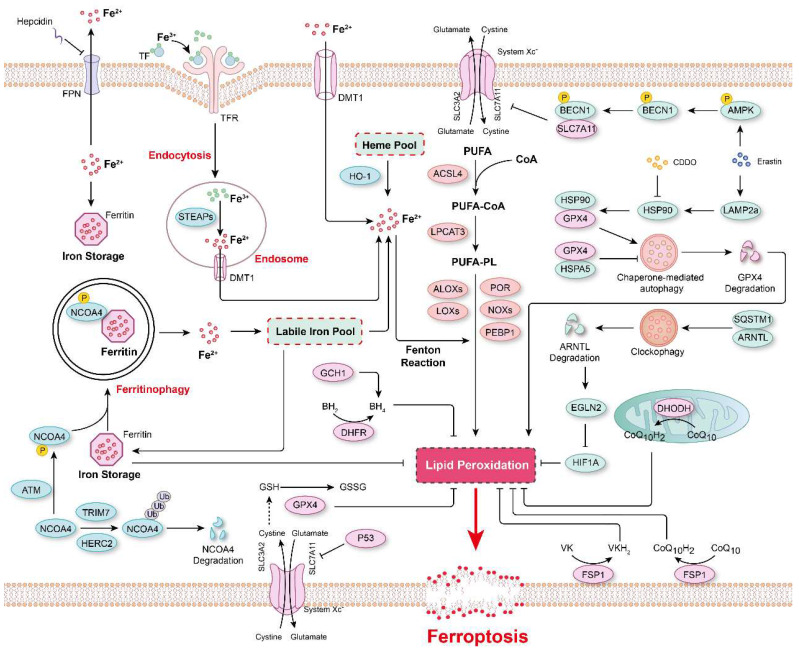
** The major mechanisms regulating ferroptosis.** The mechanisms underlying lipid peroxidation, regulatory signaling, intracellular iron storage/release and import/export, and ferritinophagy in ferroptosis. The major pathways that regulate ferroptosis include glutathione peroxidase 4 (GPX4)-glutathione (GSH), ferroptosis-suppressor-protein 1 (FSP1)-CoQ_10_, FSP1-vitamin K (VK), dihydroorotate dehydrogenase (DHODH)-CoQ, and GTP cyclohydrolase 1 (GCH1)-tetrahydrobiopterin (BH_4_).

Ferroptosis is a form of programmed cell death regulated by multiple cellular metabolic pathways, such as iron metabolism, redox homeostasis, and mitochondrial activity, and executed by phospholipid hydroperoxides (PLOOHs), a form of lipid-based reactive oxygen species (ROS)^1^, ^2^, ^13^. However, the subcellular membrane lipid peroxidation that is essential for ferroptosis has remained uncharacterized for many years. Recently, von Krusenstiern *et al.* demonstrated that various subcellular membranes undergo lipid peroxidation during ferroptosis, and the resulting lipid peroxides accumulate initially in the endoplasmic reticulum (ER) membrane and later in the plasma membrane^30^. Their results indicated that the ER membrane is a key site of lipid peroxidation during ferroptosis^30^. Moreover, at least two membrane-remodeling enzymes, lysophosphatidylcholine acyltransferase 3 (LPCAT3) and acyl-CoA synthetase long-chain family member 4 (ACSL4), have been reported to contribute to lipid peroxidation^31^, ^32^. Genetic loss or pharmacological inhibition of ACSL4 leads to a marked shift from the incorporation of long-chain polyunsaturated fatty acid (PUFA) tails to that of short-chain and monounsaturated fatty acyl (MUFA) tails into phospholipids to prevent ferroptosis^31^, ^33^, ^34^. Therefore, increasing the content of MUFAs in cells, such as that mediated via ACSL3-dependent enrichment of membranes with MUFAs, stearoyl-CoA desaturase 1 (SCD1)-mediated cellular MUFA production, and supplementation with exogenous MUFAs, has been reported to inhibit ferroptosis^35^-^37^. Furthermore, certain lipoxygenases (LOXs) have been found to be involved in ferroptosis because of their ability to directly oxygenate PUFAs and PUFA-containing lipids in biological membranes^36^, ^38^, ^39^ **(Figure 2)**. For example, arachidonate 12-lipoxygenase, 12S type (ALOX12) inactivation abrogated p53-mediated ferroptosis in cancer cells, and ALOX12 also nullified p53-dependent inhibition of tumor growth in xenograft model mice^40^. In a myocardial ischemia/reperfusion (I/R) injury model, arachidonic acid 15-lipoxygenase 1 (ALOX15) was found to be the primary mediator of ischemia-induced PUFA-phospholipid peroxidation, triggering ferroptosis and exacerbating myocardial damage^41^. In summary, these studies indicated that the composition of phospholipids in biological membranes, especially the degree of unsaturation lipids in bilayer membranes, is key for determining the vulnerability of cells to ferroptosis.

### Iron in ferroptosis

Lipid peroxidation in biological membranes is mediated by the nonenzymatic, iron-dependent Fenton reaction^13^, ^42^. Both ferrous and ferric ions are involved in the conversion of PLOOHs to the free radicals PLO• and PLOO•, which trigger damaging peroxidation chain reactions^13^, ^43^. Therefore, it is critical to maintain cellular iron homeostasis, and intracellular iron storage/release and import/export are intricately regulated **(Figure 2)**. In mammals, iron is mostly present in the form of heme, and heme oxygenase-1 (HO-1) catalyzes the degradation of heme into biliverdin, iron, and carbon monoxide^44^, ^45^. Ferric ions bind to transferrin (Tf), which is recognized by its receptor TfR and then imported into cells through TfR-mediated endocytosis^13^, ^46^, ^47^. In addition, the metal transporters DMT1 (Divalent metal transporter 1) and SLC39A14 (Solute carrier family 39 member 14) are important channels for iron import^2^, ^13^, ^48^. In contrast, Ferroportin (FPN, also known as SLC40A1) is a ferrous iron Fe(II) transporter that is critical for the release of Fe(II) from cells^49^, ^50^. Systemic iron homeostasis is regulated by hepcidin, a regulatory peptide produced mainly in the liver, and it binds to FPN, leading to the internalization and degradation of FPN^50^, ^51^. Furthermore, a prevalent FPN Q248H mutation reduces sensitivity of cells to physiological concentrations of hepcidin and prevents hepcidin-induced degradation of FPN in humans^52^, ^53^. Numerous cellular processes regulate the sensitivity of cells toward ferroptosis by altering the intracellular labile iron content, and the iron chelator deferoxamine (DFO) can completely inhibit ferroptosis^13^, ^54^, ^55^. Ferritin, a spherical iron storage protein in cells, is composed of a combination of 24 subunits of heavy-chain ferritin (ferritin-H) and light-chain ferritin (ferritin-L), which bind and sequester intracellular iron in the form of ferric ions^13^, ^56^, ^57^. Overexpression of ferritin reduces the content of intracellular labile iron and prevents lipid peroxidation and ferroptosis^57^, ^58^.

Interestingly, the protein level of ferritin is regulated via a biological process named ferritinophagy^59^ **(Figure 2)**. Nuclear receptor coactivator 4 (NCOA4) functions as a cargo receptor that mediates the delivery of iron-filled ferritin-H and ferritin-L to lysosomes via autophagy for breakdown, resulting in the release of iron^59^. An excessive rate of ferritinophagy can lead to intracellular iron overload and subsequent lipid peroxidation and ferroptosis^60^, ^61^. The Ser/Thr protein kinase ATM phosphorylates NCOA4 to enhance its interaction with ferritin, thereby increasing the ferritinophagy rate and the level of intracellular labile free iron, resulting in ferroptosis^62^. In contrast, tripartite motif-containing protein 7 (TRIM7) directly binds to and ubiquitinates NCOA4 in a K48-linked polyubiquitylation manner, facilitating the degradation of NCOA4 and reducing the ferritinophagy and ferroptosis of human glioblastoma cells^63^. Similarly, after iron repletion, the E3 ubiquitin ligase HERC2 (HECT and RLD domain-containing E3 ubiquitin protein ligase 2) interacts with NCOA4 and induces its proteasomal degradation, rendering HERC2 a negative regulator of ferritinophagy^60^.

### Autophagy in ferroptosis

In addition to ferritinophagy, upregulated microtubule-associated protein 1 light chain 3 II (LC3 II) expression and increased autophagic flux are observed during ferroptosis^64^, ^65^. Moreover, autophagy inhibitors (*e.g.,* bafilomycin A1 and chloroquine) or knockout of autophagy-related gene 3 (ATG3) and ATG13, two core contributors to autophagy, reduced cell sensitivity to ferroptosis^66^. In Erastin-induced ferroptosis, the expression of lysosome-associated membrane protein 2a (LAMP2a) is increased, which promotes heat shock protein 90 (HSP90)-dependent autophagy, which in turn mediates the degradation of GPX4^67^. Inhibiting HSP90 function with 2-amino-5-chloro-N, 3-dimethylbenzamide (CDDO) blocks GPX4 degradation and prevents cells from undergoing ferroptosis^67^. Additionally, the molecular chaperone heat shock protein family A member 5 (HSPA5) interacts with GPX4 and blocks GPX4 degradation, thus decreasing the sensitivity of cells to ferroptosis^68^. Similarly, after ferroptosis stimulation, phosphorylated AMP-activated protein kinase (AMPK) mediates BECN1 phosphorylation, leading to the formation of the BECN1-SLC7A11 complex, which blocks System Xc^-^ activity and aggravates ferroptosis^69^. Recently, a novel selective autophagy process, termed clockophagy, has been described to mediate ferroptosis through the ARNTL-EGLN2-HIF1A signaling pathway^70^. The core circadian clock protein ARNTL regulates the expression of the transcription factor HIF1A, inhibiting ferroptosis by suppressing the transcription of EGLN2^71^. The activation of clockophagy selectively degrades ARNTL through cargo receptor sequestosome 1 (SQSTM1), destabilizing HIF1A protein, and ultimately exacerbating lipid peroxidation and ferroptosis^71^. Furthermore, lipophagy, a biological process involved in the autophagic degradation of intracellular lipid droplets, has been implicated in ferroptosis^72^. Thus, some scholars have proposed that ferroptosis is an autophagy-dependent form of programmed cell death^73^, ^74^ **(Figure 2)**. Nevertheless, more evidence is necessary to confirm a causal relationship between ferroptosis and autophagy and to indicate whether the factors in these processes show synergistic relationships. Furthermore, in any causal relationship that might be established, its dependence on specific treatments will need to be determined.

### Major pathways and defense systems in ferroptosis regulation

Lipid peroxidation has been identified as a key driver of ferroptosis; thus, the redox systems play a critical role in this process. More than 80-90% of total ROS are reportedly generated by mitochondrial electron chain Complexes I and III, and NADPH after oxidation by NOXs (NADPH oxidases) is another important source of O_2_•^-^ and H_2_O_2_ producing-proteins in signal transduction^75^, ^76^. Hydrogen peroxide is converted into highly toxic hydroxyl free radicals in the presence of ferrous ions via the Fenton reaction^76^. Multiple peroxidase system enzymes, such as glutathione peroxidase (GPX), catalase, NADH peroxidase, thioredoxin (TRX), and peroxiredoxin (PRX), are involved in converting H_2_O_2_ into H_2_O^75^, ^77^. Additionally, ubiquinone (also known as coenzyme Q) is a ubiquitous lipid-soluble redox cofactor that primarily transfers electrons and protons across the inner mitochondrial membrane^78^. Therefore, any disorder in these redox/defense systems accelerates lipid peroxidation and facilitates the onset of ferroptosis **(Figure 2)**.

GPX4-GSH was the first known redox system to be implicated in the regulation of ferroptosis^79^. By using targeted metabolomic profiling, Yang* et al.* demonstrated that depletion of glutathione causes the inactivation of GPXs in response to ferroptosis inducers. Furthermore, GPX4 overexpression inhibits the lethality of 12 ferroptosis inducers, which indicates that GPX4 is a central regulator of ferroptosis^79^. Several GPX4 inhibitors, including RSL3, ML162 and ML210, have been shown to augment ferroptosis in many cell types and cell lines^80^-^82^. In both RSL3- and ML162-resistant cells, ACSL4 and LPCAT3 have been shown to be significantly enriched for gene trap insertions, indicating that ACSL4 and LPCAT3 are indispensable for the execution of GPX4 inhibition-induced ferroptosis^83^. The xCT complex, composed of solute carrier family 7 member 11 (SLC7A11) and SLC3A2, mediates the exchange of cystine and glutamate across the plasma membrane, promoting GSH synthesis^84^. Therefore, cystine deficiency or the inhibition of xCT function leads to impaired GSH synthesis and promotes ferroptosis^3^, ^4^, ^85^-^88^. P53, a tumor suppressor, has been reported to inhibit cystine uptake and sensitize cells to ferroptosis by downregulating the expression of SLC7A11^88^. SLC7A11 binds directly to ALOX12 and inhibits its lipoxygenase activity^40^, which offsets P53-mediated ferroptosis induced by ROS stress and reverses P53-dependent tumor growth suppression in xenograft models^40^. In contrast, cancer cells with high levels of SLC7A11 (SLC7A11^high^) have been observed to accumulate intracellular cystine when glucose levels are low, draining the cellular NADPH pool and leading to the aberrant accumulation of intracellular disulfides and subsequently to disulfidptosis, a previously uncharacterized form of cell death that differs from apoptosis and ferroptosis^89^, ^90^. Further investigation revealed that disulfidptosis is inhibited by inactivation of the WAVE regulatory complex, while it is facilitated by the constitutive activation of Rac^90^. These studies demonstrated that maintaining cystine homeostasis within cells is essential for cell survival, as cystine deficiency causes ferroptosis, and excessive cystine accumulation leads to disulfidptosis.

Although xCT-GPX4-GSH is considered to be the primary mechanism that prevents ferroptosis, inhibition of GPX4 or GPX4 knockout fails to trigger ferroptosis in certain cancer cell lines, suggesting alternative resistance mechanisms^79^, ^91^-^93^. Therefore, to identify genes complementing GPX4 loss, Doll* et al.* generated a cDNA expression library derived from MCF7 cells (a ferroptosis-resistant cell line) and, through screening, discovered that the pro-apoptotic gene AIFM2 (apoptosis inducing factor mitochondria-associated 2) is a novel anti-ferroptotic gene, which they renamed “ferroptosis-suppressor-protein 1” (FSP1)^93^. A back-to-back study using a synthetic lethality strategy with CRISPR-Cas9 also shown FSP1 to be a potent ferroptosis-resistance factor in U-2 OS osteosarcoma cells treated with the GPX4 inhibitor RSL3^92^. Furthermore, the myristoylation of FSP1 and its subsequent plasma membrane localization were critical for reducing non-mitochondrial CoQ_10_, which is an antioxidant that prevents lipid peroxidation and ferroptosis^92^. These studies indicated that FSP1-CoQ_10_ is a novel anti-ferroptotic pathway that parallels to the canonical xCT-GPX4-GSH pathway. In addition, an FSP1-dependent noncanonical VK cycle has recently been reported to function as a ferroptosis suppressor, and FSP1 efficiently reduced VK to its hydroquinone VKH_2_ form, which function as a potent radical-trapping antioxidant to inhibit lipid peroxidation^18^. In addition to CoQ_10_ at the plasma membrane, mitochondrial CoQ_10_ is also reduced into CoQH_2_ in the mitochondrial inner membrane via the action of DHODH to attenuate ferroptosis^17^. In an RSL3-, imidazole ketone erastin (IKE)-, or GPX4 knockout-induced ferroptosis model, CRISPR activation screening revealed that GCH1, the rate-limiting enzyme for 6(*R*)-L-erythro-5,6,7,8-tetrahydrobiopterin (BH_4_) synthesis, is a gene that antagonizes ferroptotic cell death^94^. BH_4_ is a critical cofactor for nitric oxide synthases (NOS); under BH_4_-deficient conditions, superoxide is generated instead of NO, increasing the susceptibility of cells to ferroptosis^95^. Furthermore, it is reported that IKE-induced high BH_4_ levels led to CoQ_10_ synthesis, thus alleviating oxidative damage and preventing ferroptosis^95^. Interestingly, Wu* et al.* found that ferroptosis was non-cell-autonomously regulated by E-cadherin-mediated intercellular interactions, which repressed ferroptosis by activating intracellular NF2 (neurofibromin 2)-YAP (Yes1-associated transcriptional regulator) signaling^96^. In addition, many other mechanisms regulating ferroptosis have been revealed, such as the AMPK-mediated anti-ferroptotic effect of energy stress^97^ and glutamate-cysteine ligase catalytic subunit (GCLC)-conferred protection against ferroptosis mediated by its contribution to maintaining glutamate homeostasis^86^.

More importantly, epigenetic modifications are essential regulatory mechanisms for biological processes in the body, and recent studies have shown that ferroptosis is regulated by various epigenetic modifications, including DNA methylation, the RNA m^6^A modification and protein methylation^3^, ^8^, ^98^, ^99^. Research in this field is rapidly producing advancements, with new epigenetic mechanisms in ferroptosis being uncovered at an ever-increasing rate. Therefore, it is essential to review and discuss relevant studies.

## DNA methylation in ferroptosis

**Figure 3:**
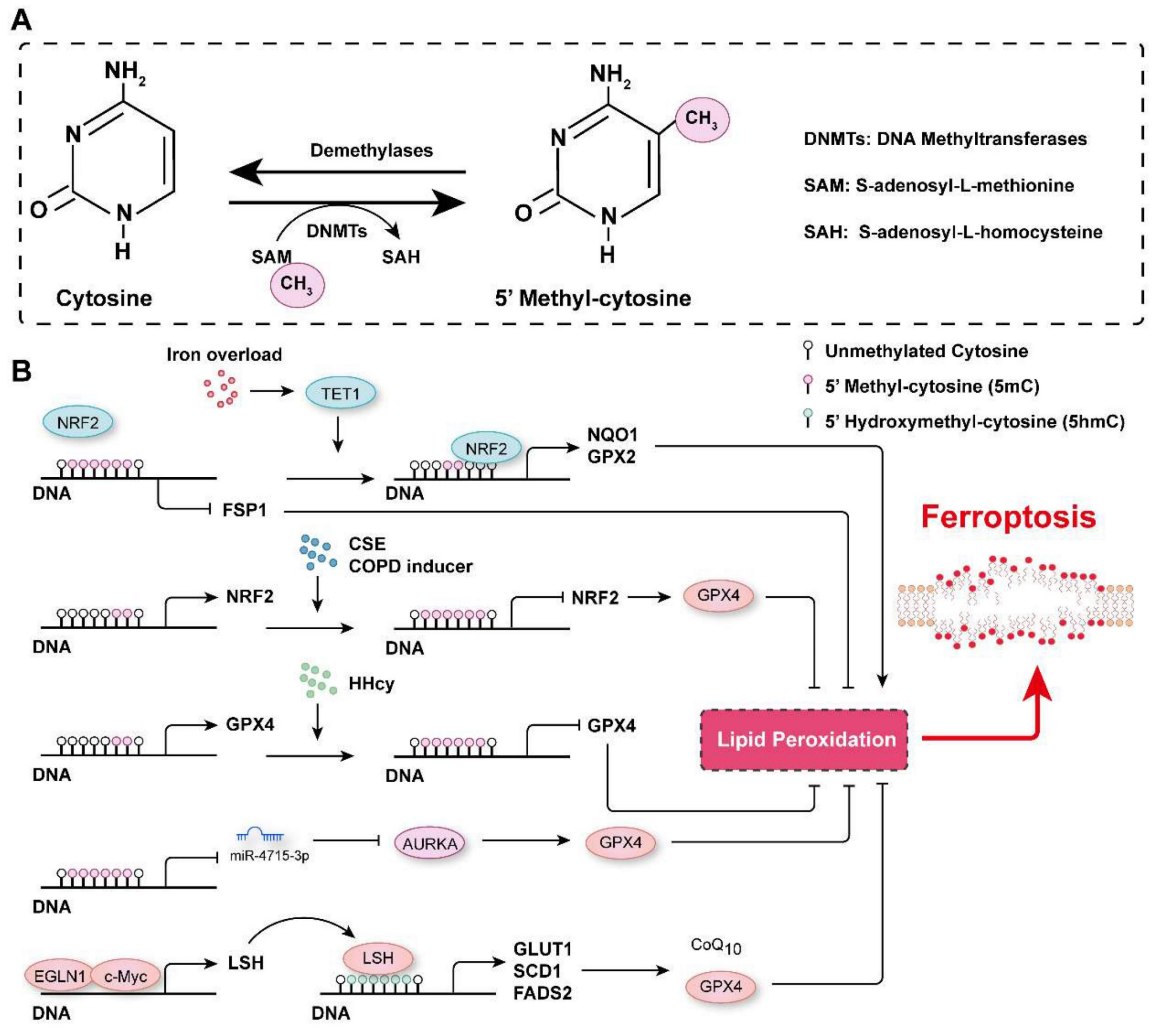
** DNA methylation in the regulation of ferroptosis. A.** Schematic representation of DNA methylation. DNA is usually methylated on the fifth position of the cytosine base in mammals, generating 5-methyl-cytosine (5mC), which is catalyzed by DNMTs, and the methyl group is removed by demethylases. **B.** The mechanisms related to DNA methylation involved in the regulation of ferroptosis. CSE: Cigarette smoke extract; COPD: Chronic obstructive pulmonary disease; (HHcy): Hyperhomocysteinemia.

DNA methylation is an epigenetic modification that controls gene expression, typically occurring at the fifth position of the cytosine base in mammals to generate 5-methyl-cytosine (5mC). This modification is usually observed on cytosine-phosphate-guanine (CpG) dinucleotides, and approximately 60-90% of CpGs in the genome are methylated^100^. DNA methylation is dynamically regulated by DNA methyltransferases and demethylases **(Figure 3A)**. DNA methyltransferase 3A (DNMT3A), DNMT3B and DNMT3C are critical for *de novo* DNA methylation, which is maintained by DNMT1 through DNA replication^101^, ^102^. In contrast, ten-eleven translocation (TET) enzymes actively remove methyl groups from DNA and successively oxidize 5mC to hydroxymethyl-cytosine (5hmC), formyl-cytosine (5fC) and carboxyl-cytosine (5caC)^100^, ^103^-^105^. DNA methylation marks have been reported to be involved in many biological processes and diseases^106^, ^107^, and their roles in ferroptosis regulation has recently been highlighted.

By analyzing the data in The Cancer Genome Atlas, Liu *et al.* revealed that DNA methylation and somatic copy number alterations (SCNAs) may contribute to the differential expression of most ferroptosis regulator genes (FRGs) in tumors^108^. They further established the ferroptosis potential index (FPI) and found that, compared with normal tissues, most cancer cells were characterized with a high FPI and that a high FPI predicted poor prognosis for several tumors^108^. Similarly, many ferroptosis-associated DNA methylation signatures have been developed to predict the prognosis for various tumors, such as glioblastoma multiforme (GBM), head and neck squamous cell carcinoma (HNSCC), cutaneous melanoma (CM), and lung squamous cell carcinoma (LUSC)^109^-^112^. DNA methylation of FRGs is involved in the regulation of their expression. For instance, analyzing the most recent TCGA multidimensional omics data revealed that multiple iron-related genes (*e.g.,* scavenger receptor class A member 5 (SCARA5), erythroferrone (ERFE), lipocalin 2, transferrin receptor 2 (TFR2), solute carrier family 11 member 1 (SLC11A1), and cytochrome B reductase 1 (CYBRD1)) were dysregulated in different cancers, and this outcomes may be associated with aberrant DNA methylation^112^. Hypomethylation of the epigenome and increased TET1 expression have been observed in colonocytes subjected to continuous iron exposure^113^. Moreover, iron overload-induced DNA demethylation promotes the expression of nuclear factor erythroid 2-related factor 2 (NRF2) targets, such as NQO1 (NAD(P)H quinone dehydrogenase 1) and GPX2, which subsequently contribute to cellular lipid peroxidation and ferroptosis^113^. It has been reported that EGLN2 (Egl-9 family hypoxia inducible factor 2) is a candidate driver of iron chelation-mediated inhibition of cell death, and the hydroxylase activity of EGLNs is iron-dependent^114^, ^115^. In lung cancer cells, EGLN1 and c-Myc are recruited to the promoter of lymphoid-specific helicase (LSH) at two hypoxia inducible factor-1α (HIF-1α) binding sites, which directly facilitates the expression of LSH by inhibiting HIF-1α^115^. LSH, a DNA methylation modifier and a reader of 5-hmC, increases the 5-hmC levels at the promoters of the lipid metabolism-associated gene GLUT1 (glucose transporter type 1) and fatty acid desaturase genes SCD1 (stearoyl-CoA desaturase 1) and FADS2 (fatty acid desaturase 2) to activate their expression in H358 cells and PC9 cells^115^, ^116^. Activation of SCD1 increased antioxidant CoQ_10_, concomitantly increased the number of unsaturated fatty acyl chains in membrane phospholipids and decreased the number of long-chain saturated ceramides, which protected cells from ferroptosis^37^. In contrast, inhibition of SCD1/FADS2 directly downregulated the expression of GPX4 and reduced the ratio of GSH/GSSG, thereby accelerating iron-mediated lipid peroxidation, mitochondrial dysfunction and ferroptosis^117^. Phosphatidylethanolamine (PE)-linked arachidonic acid (AA) and adrenic acid (AdA) are substrates for lipid peroxidation^118^. Lee *et al.* demonstrated that FADS1 and elongation of very long-chain fatty acid protein 5 (ELOVL5) were required to maintain intracellular levels of AA and AdA, and the expression of FADS1 and ELOVL5 was found to be frequently inhibited by an increase in the DNA methylation marks on at their promoter/enhancer regions in intestinal-type gastric cancer cells (GCs), which resist ferroptosis^118^. In contrast, the expression of FADS1 and ELOVL5 was upregulated in mesenchymal-type GCs, which are sensitive to ferroptosis^118^.

GPX4 has been reported to be overexpressed in cancer tissues compared to normal tissues and to be associated with low levels of DNA methylation and enrichment of H3K4me3 and H3K27ac marks at its promoter, suggesting that epigenetic regulation may be involved in its aberrant overexpression^22^. Hyperhomocysteinemia (HHcy), a risk factor for cardiovascular diseases, neurological disorders, and musculoskeletal system dysfunction, has been reported to promote GPX4 methylation, leading to oxidative stress and ferroptosis^119^. In chronic obstructive pulmonary disease (COPD) patients and human bronchial epithelial (HBE) cells treated with cigarette smoke extract (CSE), GPX4 expression is decreased^21^. Furthermore, *Nrf2* promoter hypermethylation is observed in both COPD patients and CSE-treated HBE cells, resulting in NRF2 downregulation, inhibited GPX4 expression and ferroptosis occurrence^21^. Gomaa *et al.* found increased DNA methylation levels on several CpG nucleotides upstream of miR-4715-3p in upper gastrointestinal adenocarcinoma (UGC) tissue samples, which binds to the 3' untranslated region (3' UTR) of Aurora kinase A (AURKA) to inhibit its expression^120^. Moreover, reconstitution of miR-4715-3p or inhibition of AURKA suppressed GPX4 expression to induce ferroptosis^120^. xCT has been reported to be involved in maintaining intracellular GSH levels, redox balance and anti-ferroptotic effects^121^. The stability of xCT is regulated by the MUC1-C (Mucin 1 C-terminal subunit)/CD44v (CD44 variant) complex, which directly interacts with xCT to promote its stability and control GSH levels^122^. Moreover, the expression of MUC1 is controlled by histone and DNA methylation on its promoter^122^. Additionally, the promoter of the *Fsp1* gene has been shown to be hypermethylated in T- and B- acute lymphoblastic leukemia cell lines and patient samples, in which it suppressed FSP1 expression and promoted ferroptosis^20^. DNA methylation of the *Fsp1* promoter prevented NRF2 from binding to its promoter and inhibited transcription^20^, ^123^. In addition, DNA methylation of many other genes has been reported to contribute to ferroptosis regulation^98^, ^124^-^128^. For example, embryonic lethal-abnormal vision-like protein 1 (ELAVL1), a well characterized RNA-binding protein that promotes target mRNA stability, has been found to bind to the 3'UTR of DNMT3B mRNA, thereby facilitating its expression during cerebral ischemia/reperfusion (I/R) injury. Increased DNMT3B levels accelerate the DNA methylation of the *Pink1* (PTEN induced kinase 1) promoter, inhibiting PINK1 expression and promoting ferroptosis and brain damage^129^.

Studies have indicated that DNA methylation is critical for ferroptosis, as these marks regulate the expression of ferroptosis-related genes **(Figure 3B)**. Generally, hypermethylation in the promoter or transcriptional start site or hypomethylation in the coding sequence of a gene suppresses the expression of that gene. However, it should be noted that the expression of a methylated gene can be increased when either the coding sequence is hypermethylated or the promoter or transcriptional start site is hypomethylated^130^. Thus, DNA methylation in coding regions needs to be further studied, especially when no difference in overall DNA methylation is identified, and the distribution of DNA methylation throughout the genome needs to characterized.

## The RNA m^6^A modification in ferroptosis

**Figure 4:**
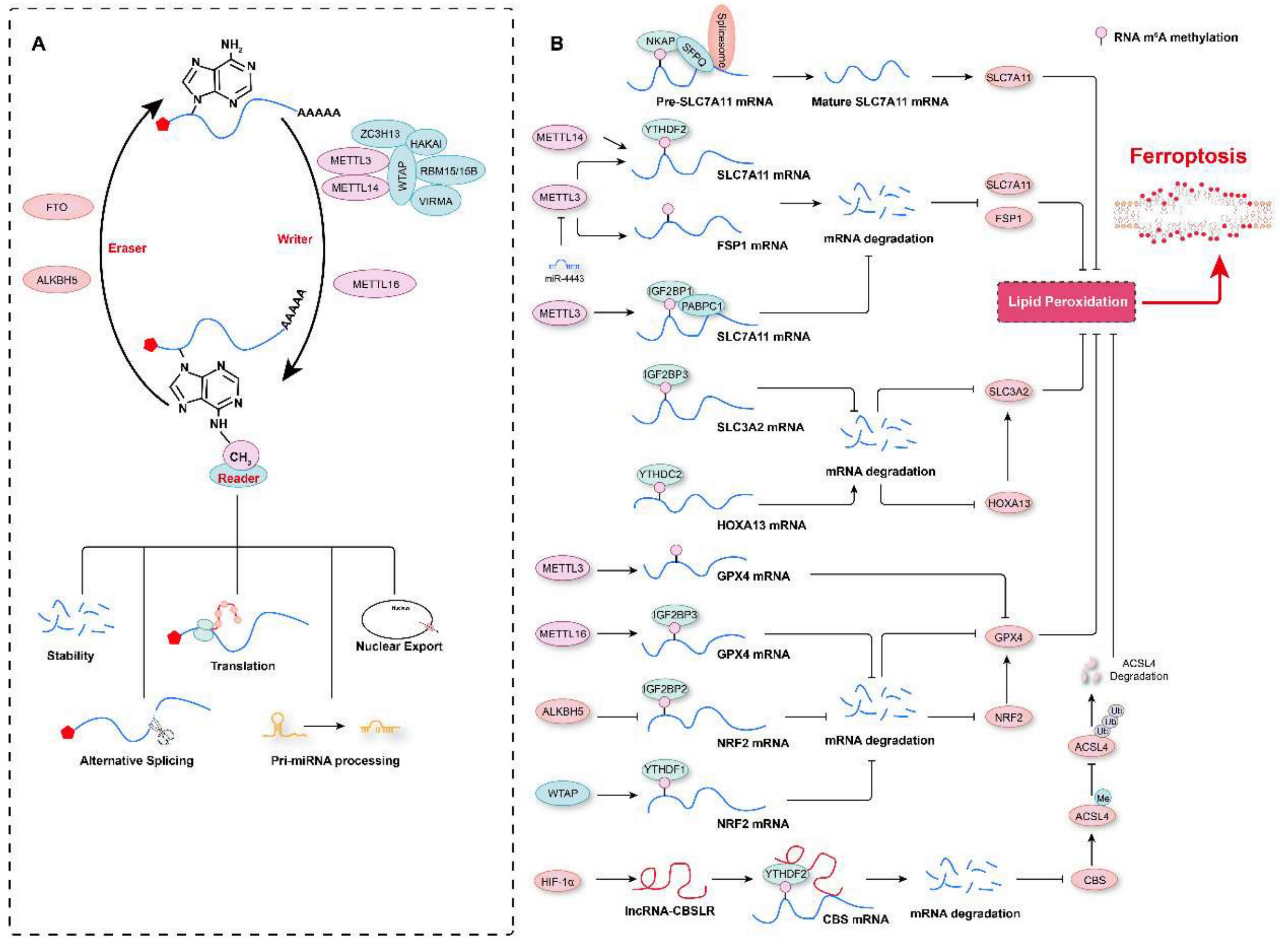
** RNA m^6^A modification in the regulation of ferroptosis. A.** Schematic representation of RNA m^6^A modification. RNA m^6^A marks are deposited by “writers”, and methyltransferase-like 3 (METTL3), METTL14, and METTL16 are the major methyltransferases. In contrast, an RNA m^6^A methyl group is removed by “erasers”, such as fat mass and obesity-associated protein (FTO) and AlkB homolog 5 (ALKBH5). m^6^A-modified RNA is recognized by “readers”, which determine the fate of the RNA, such as degradation, translation, alternative splicing, pri-miRNA processing, or nuclear export. **B.** The RNA m^6^A modification contributes to ferroptosis regulation by affecting the stability of mRNAs or their alternative splicing.

To date, more than 100 RNA modifications, including N^6^-methyladenosine (m^6^A), 5-methylcytosine (m^5^C), N^1^-methyladenosine (m^1^A), N^3^-methylcytosine (m^3^C), N^7^-methylguanosine (m^7^G), and pseudouridine (Ψ), have been identified^131^-^133^. Of these marks, m^6^A is one of the most abundant modifications of cellular RNA^134^. It was first discovered by two independent research groups in 1974 in poly(A) RNA fractions^135^, ^136^. RNA m^6^A marks are usually located in 3' UTRs, although there are also a few m^6^A sites located in regions surrounding start codons and 5' UTRs^137^-^139^. RNA m^6^A marks are deposited by a complex comprising methyltransferase-like 3 (METTL3), METTL14, Wilms' tumor 1-associated protein (WTAP), and KIAA1429, with METTL3 being the major subunit with the methyltransferase activity and acting as the “writer”^134^, ^140^, ^141^. In contrast, fat mass and obesity-associated protein (FTO) and AlkB homolog 5 (ALKBH5) have been found to have demethylase activity, functioning as “erasers” of the m^6^A mark^142^, ^143^. m^6^A-methylated RNA is recognized by “readers”, such as YT521-B homology (YTH) domain family proteins (YTHDF1, YTHDF2, YTHDF3, and YTHDC1) and heterogeneous nuclear ribonucleoprotein family proteins (HNRNPA2B1 and HNRNPC), which determine the fate of the marked RNA, such as degradation, translation or splicing^144^-^147^ **(Figure 4A)**. Furthermore, the role of the RNA m^6^A modification in many biological processes and diseases has been elucidated in the past decade; these processes include autophagy, cell proliferation, stem-cell renewal and differentiation, and the diseases include tumorigenesis, and cardiovascular diseases^139^, ^146^, ^148^, ^149^. In the past three years, several studies have shown that RNA m^6^A marks are involved in the regulation of ferroptosis and thus affects the progression of diseases such as tumor growth and acute kidney injury^133^, ^150^, ^151^ and that m^6^A modification regulators used in combination with ferroptosis-related genes can be diagnostic or prognostic markers for tumors in humans^130^, ^152^-^155^.

Multiple m^6^A modification regulators have been shown to be aberrantly expressed in various types of tumors, and a prediction model comprising these regulators and ferroptosis-associated genes has been shown to effectively reflect the progression and prognosis of these tumor patients^23^, ^156^-^159^. Recently, our research revealed that compared to aortic samples without aortic dissection (non-AD), higher levels of METTL3 and METTL14 protein, but lower levels of FTO were detected in aortic samples with Stanford type A aortic dissection (TAAD)^3^. Moreover, METTL3 protein levels were inversely correlated with the levels of ferroptosis modulators SLC7A11 and FSP1 in human aortas. METTL3 has also been shown to repress SLC7A11 and FSP1 expression by promoting decay of their mRNAs in human aortic smooth muscle cells (HASMCs). Furthermore, METTL3 accelerated IKE- and cystine deprivation-induced ferroptosis of HASMCs^3^. Similarly, in lipopolysaccharide (LPS)-induced cardiomyocytes, METTL3 deficiency inhibited LPS-induced ferroptosis by upregulating the expression of SLC7A11 in an m^6^A methylation-dependent manner^160^. METTL3 has been found to promote m^6^A modification of SLC7A11 mRNAs, which were then recognized by YTHDF2 to mediate their degradation^160^. Similar to METTL3, METTL14, another m^6^A methyltransferase, installs m^6^A modification on the 5'UTR of SLC7A11 mRNAs to facilitate their degradation in a YTHDF2-dependent manner, and hypoxia induces the downregulation of METTL14 by regulating HIF-1α levels, thus protecting hepatocellular carcinoma (HCC) cells from ferroptosis^161^. In contrast, in hepatoblastoma cells, IGF2 mRNA-binding protein 1 (IGF2BP1) has been identified as the m^6^A reader, and it was shown to compete with the BTG2/CCR4-NOT complex to bind PABPC1, thereby inhibiting SLC7A11 mRNA deadenylation and promoting its stability, resulting in increased SLC7A11 expression and the eventual inhibition of ferroptosis^24^. Intriguingly, the m^6^A demethylase FTO has been shown to inhibit SLC7A11 expression and thus trigger ferroptosis in an m^6^A-independent manner, attenuating the development of papillary thyroid carcinoma^162^. In addition to regulating SLC7A11 mRNA stability, the m^6^A modification has also been observed to participate in ferroptosis by affecting the mRNA splicing of SLC7A11^163^. For example, NF-κB-activating protein (NKAP) has been discovered to directly bind to m^6^A-methylated SLC7A11 mRNA and then recruit the splicing factor proline and glutamine-rich (SFPQ), which recognize the splice site and thus promotes SLC7A11 mRNA splicing and maturation, ultimately preventing the ferroptosis of glioblastoma cells^163^. These studies indicate that the m^6^A modification of SLC7A11 mRNA is critical for its expression regulation and ferroptosis. However, some results describing the impact of m^6^A on SLC7A11 expression are debated, and further investigation is needed. For example, more studies need to be conducted to explore the differences in m^6^A methylation sites in SLC7A11 mRNAs under different conditions, and these marks deposited on different sequence may lead to different fates of mRNAs after being recognized by different readers.

In addition to SLC7A11 mRNAs, the mRNAs of its partner SLC3A2 is also regulated by the m^6^A methylation. For example, in patients with lung adenocarcinoma (LUAD), the m^6^A reader insulin-like growth factor 2 mRNA-binding protein 3 (IGF2BP3) is highly expressed and binds to m^6^A-marked mRNAs encoding anti-ferroptosis regulators (*e.g.,* SLC3A2, GPX4, ACSL3, and ferritin heavy chain 1 (FTH1)) to increase the stability of these mRNAs^164^. In contrast, Ma *et al.* demonstrated that SLC3A2 is not directly regulated by the m^6^A modification of its mRNA but is positively regulated by the transcription factor homeobox A13 (HOXA13), whose mRNAs are m^6^A-methylated on its 3'UTR, and this modification is recognized by YTHDC2, which causes the degradation of HOXA13 mRNAs in LUAD cells^165^. These results indicate that increasing YTHDC2 expression may be an alternative treatment option for LUAD because it facilitates ferroptosis via the downregulation of SLC3A2. Thus, the expression of SLC3A2 in LUAD cells may be regulated by in both an m^6^A methylation-dependent and -independent manner. GPX4 is another critical ferroptosis regulator^166^, ^167^. In samples obtained from breast cancer patients, METTL16 is overexpressed, which increases the rate of m^6^A modification on GPX4 mRNAs, increasing their stability and resulting in upregulation of GPX4. Enhanced GPX4 expression accelerates the proliferation and inhibits the ferroptosis of breast cancer cells^168^. However, in sepsis-associated acute lung injury (SI-ALI), neutrophil extracellular traps (NETs) activate the TLR9/MyD88/NF-κB pathway to upregulate METTL3 expression, which induces the m^6^A methylation of GPX4 mRNAs, inhibiting GPX4 expression and increasing the ferroptosis of alveolar epithelial cells^169^. It is crucial to elucidate the differences in GPX4 m^6^A marks on the regulation of GPX4 expression under different pathological conditions. Do different methylation sites or the activity of different readers lead to different fates of methylated mRNAs?

With research advances in this field, the mRNAs of multiple ferroptosis regulators have been shown to modified with m^6^A marks. For example, in non-small cell lung carcinoma (NSCLC) cells, exosomal miR-4443 targets METTL3 expression and negatively regulates METTL3-mediated m^6^A modifications on FSP1 and ferroptosis^170^. ALKBH5-mediated m^6^A demethylation at two m^6^A residues in the 3'UTR of NFE2L2/NRF2 mRNAs, which are recognized by IGF2BP2, contributes to reduced mRNA stability and decreased NFE2L2/NRF2 expression in hypopharyngeal squamous cell carcinoma cells^171^. WTAP can deposit m^6^A marks on the 3'UTR of NRF2 mRNA, and these marks are read by YTHDF1, enhancing the stability of NRF2 mRNA and inhibiting ferroptosis in bladder cancer cells^172^. In addition, autophagy is an important mechanism for regulating ferroptosis, and the m^6^A modification on the mRNAs of the autophagy-related gene BECN1 results in these m^6^A-marked mRNAs resistant to degradation after recognition by YTHDF1, contributing to dihydroartemisinin-induced hepatic stellate cell ferroptosis and conferring protection against liver fibrosis^173^. Hypoxia can also induce HIF-1α-mediated expression of lncRNA-CBSLR, which recruits YTHDF2 to read m^6^A-modified CBS (cystathionine β-synthase) mRNAs, leading to decreased mRNA stability and reduced CBS expression. Decreased CBS results in reduced methylation of the ACSL4 protein and facilitates polyubiquitination and degradation of ACSL4 via the ubiquitination-proteasome pathway, ultimately protecting gastric cancer against ferroptosis^99^.

Therefore, these aforementioned studies suggest that the RNA m^6^A modification is involved in ferroptosis by regulating the mRNA stability and protein expression of multiple ferroptosis-related genes **(Figure 4B)**. However, there are still at least three scientific questions that must be answered. First, what are the mechanisms underlying the RNA m^6^A modifications involved in ferroptosis? Second, what are the mechanisms by which specific m^6^A modification sites are recognized by different readers, which determine different fates of mRNAs after ferroptosis inducers treatment? Third, does targeting m^6^A marks to regulate ferroptosis has potential as a treatment for related diseases?

## Protein methylation in ferroptosis

**Figure 5:**
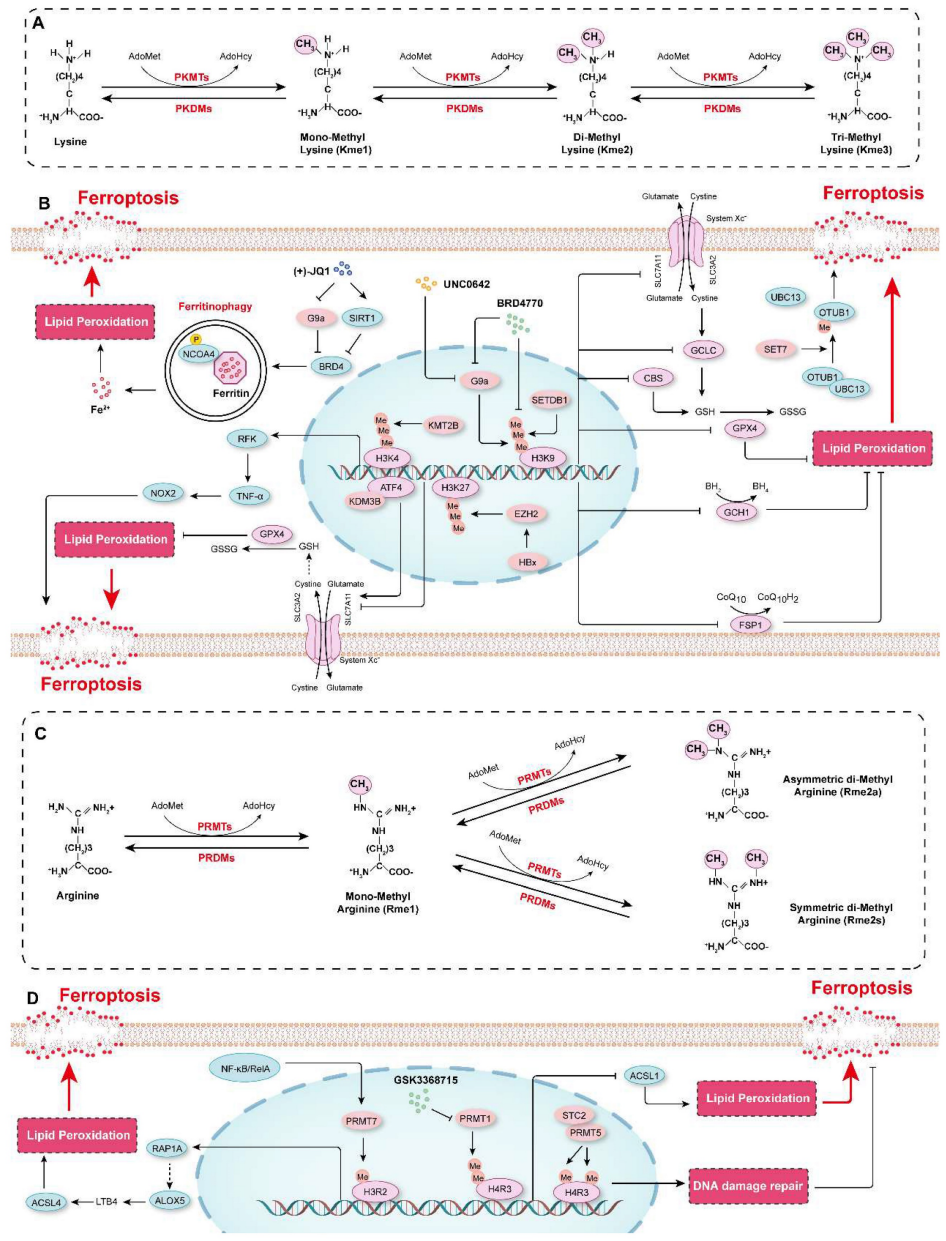
** Protein methylation in the regulation of ferroptosis. A.** Schematic diagram showing protein methylation on lysine residues. Lysine residues in protein can be methylated by lysine methyltransferases (PKMTs) with S-adenosyl-L-methionine (AdoMet) functioning as the primary methyl group donor. Lysine can be monomethylated (Kme1), dimethylated (Kme2) and trimethylated (Kme3), and these modifications are reversible, and marks can be erased by lysine demethylases (PKDMs). **B.** The mechanisms by which lysine methylation regulates ferroptosis.** C.** Schematic diagram showing protein methylation on arginine residues. Arginine residues in proteins can be monomethylated (Rme1), and symmetrically dimethylated (Rme2s) or asymmetrically dimethylated (Rme2a) by arginine methyltransferases (PRMTs), and they are demethylated by arginine demethylases (PRDMs). **D.** The molecular mechanisms by which arginine methylation regulates ferroptosis.

Protein methylation occurs mainly on lysine and arginine residues, and protein lysine methyltransferases (PKMTs or PLMTs) and protein arginine methyltransferases (PRMTs) are responsible for the addition of methyl groups to these residues^174^, ^175^. Lysine can be mono-, di-, or trimethylated (me1, me2 or me3, respectively), while arginine can be mono-, symmetrically dimethylated, or asymmetrically dimethylated (me1, me2s or me2a, respectively)^176^, ^177^** (Figure 5A and 5C)**. Histone methylation is the most common type of protein methylation, such as methylation of H3K27, H3K4, H3K9, H3K36, H4K20, and H4R3^178^-^183^. In recent years, a variety of non-histone proteins have also been shown to be methylated, such as P53 and AKT^184^-^186^. Conversely, methyl groups can be removed from different residues on proteins by demethylases^187^-^190^. Protein methylation has been implicated in many biological processes (*e.g.*, programmed cell death, proliferation, and autophagy) and diseases (*e.g.*, cardiovascular diseases, cancers, and mental health disorders)^4^, ^181^-^183^, ^191^, ^192^. Recently, the role of protein methylation in ferroptosis has been increasingly recognized and highlighted^193^.

Our recent studies showed that compared to those in control cells, the protein levels of H3K9me1, H3K9me2, and H3K9me3 were increased in HASMCs treated with ferroptosis inducers (cystine deprivation, IKE, or RSL3). Further screening of the effects of several histone methyltransferase inhibitors on ferroptosis revealed that BRD4770, an inhibitor of H3K9me1/2/3, significantly inhibited ferroptosis in HASMCs, and its effect was comparable to that of Fer-1, a classical ferroptosis inhibitor. More importantly, BRD4770 delayed the progression of β-aminopropionitrile monofumarate (BAPN)-induced aortic dissection in mice by inhibiting the inflammatory response, lipid peroxidation, and ferroptosis^4^. BRD4770 had been previously reported to be an inhibitor of G9a^194^. Our findings thus suggest that G9a may be involved in the regulation of ferroptosis. Rothammer* et al.* showed that the level of H3K9me2 was significantly increased in humans with multiple sclerosis and in a murine experimental autoimmune encephalomyelitis model. G9a suppressed anti-ferroptotic genes (GPX4, CBS and GCLC), reduced intracellular GSH levels, triggering ferroptosis and resulting in neuronal damage, while targeting G9a by its inhibitor UNC0642 alleviated inflammation-induced neurodegeneration^195^. On the contrary, lysine demethylase 3B (KDM3B) increased the expression of SLC7A11 by interacting with activating transcription factor 4 (ATF4) to inhibit erastin-induced ferroptosis^196^. (+)-JQ1, an inhibitor of bromodomain-containing 4 (BRD4), suppressed the expression of the ferroptosis-associated genes GPX4, SLC7A11, and SLC3A2 by repressing the expression of G9a while simultaneously promoting the expression of histone deacetylase Sirtuin 1 (SIRT1). Subsequently, (+)-JQ1 promoted ferritinophagy to increase iron levels, which induced ferroptosis in cancer cells^197^. SET domain bifurcated 1 (SETDB1, also known as ESET or KMT1E) is another important methyltransferase that methylates H3K9^198^. SETDB1 deficiency clearly promoted the acquisition of the TGF-β-induced mesenchymal phenotype but reduced the expression of E-cadherin in human alveolar epithelial cells by catalyzing the deposition of H3K9me3 on *Snai1*, which inhibited the protein expression of Snai1, the main transcription factor that initiates the epithelial-mesenchymal transition (EMT). Overexpression of SETDB1 inhibited the EMT and caused ferroptosis, preventing pulmonary fibrosis^199^. These studies indicate that H3K9 methylation is a key epigenetic mechanism that regulates ferroptosis and that targeting H3K9 methylation regulators *(e.g.*, with BRD4770) significantly inhibits ferroptosis and is expected to be a potential treatment for degenerative diseases characterized by cell loss (*e.g.,* aortic dissection). On the other hand, enhancing H3K9 methylation can treat the diseases with excessive cell proliferation such as tumors by promoting ferroptosis.

H3K27 methylation is an important histone mark that suppresses gene expression and is primarily catalyzed by enhancer of zeste homolog 2 (EZH2)^182^, ^200^, ^201^. It has been reported that the hepatitis B virus X protein (HBx) facilitated D-GalN-induced hepatotoxicity and ferroptosis by inhibiting the expression of SLC7A11 through the EZH2-mediated H3K27me3 modification^202^. In contrast, Yu *et al.* demonstrated that EZH2 mitigated the ferroptosis of tongue squamous cell carcinoma cells by suppressing miR-125b-5p but upregulating SLC7A11 expression^203^. Additionally, GSK-J4, a dual inhibitor of histone lysine demethylase 6A/6B that prevents H3K27 demethylation, can suppress palmitic acid (PA)-induced hypersensitivity to ferroptosis by inhibiting ACSL4 expression and lipid peroxidation in cardiomyocytes^204^. KMT2B activated the transcription of riboflavin kinase (RFK) by enhancing the trimethylation of H3K4, which subsequently accelerated tumor necrosis factor-α (TNF-α)/NADPH oxidase 2 (NOX2) axis activation to promote ferroptosis during myocardial ischemia/reperfusion injury^205^.

In contrast, SET7 attenuated the inhibitory effect of OTUB1 (OTU domain-containing ubiquitin aldehyde-binding protein 1) on cystine starvation/erastin-induced ferroptosis by directly interacting with non-histone OTUB1 to catalyze its methylation on lysine 122. Interestingly, this effect is not achieved by affecting the deubiquitinase (DUB) activity of OTUB1 but by impairing its noncanonical activity, binding to the E2 enzyme UBC13^206^. Furthermore, menin-mixed-lineage leukemia (MLL) inhibitors, such as MI-463, induced ferroptosis in several cancer cell lines^207^.

In addition to lysine methylation, arginine methylation also plays a critical role in ferroptosis. For example, protein arginine methyltransferase 1 (PRMT1) has been reported to function as a suppressor of ferroptosis by increasing the level of asymmetrically dimethylated histone H4 on Arg 3 (H4R3me2a) to downregulate the expression of ACSL1 and then further decrease the lipid peroxidation^208^. Moreover, the combination of the Type I PRMT inhibitor GSK3368715 and ferroptosis inducers (e.g., RSL3) shows potential as a treatment of acute myeloid leukemia^208^. Stanniocalcin 2 (STC2) can interact with and activate PRMT5, which catalyzes symmetric dimethylation of histone H4 on Arg 3 (H4R3me2s) to induce radioresistance by activating DNA damage repair, but inhibits ferroptosis in esophageal squamous cell carcinoma^209^. The expression of PRMT7 was induced by the activation of NF-κB/RelA in macrophages in lung tissue characterized by COPD^210^. Elevated PRMT7 caused an increase in the levels of H3R2me1 at the regulatory elements of Ras-related protein Rap-1A (RAP1A), thereby promoting RAP1A expression and accelerating the adhesion and migration of monocytes. Furthermore, accumulation of monocyte-derived macrophages resulted in the overexpression of ALOX5 and the accumulation of its metabolite LTB4, which activates the expression of ACSL4 to accelerate ferroptosis and tissue injury in COPD^210^.

These studies have indicated that protein methylation, such as H3K9 and H3K27 methylation, is a critical epigenetic mechanism in the regulation of ferroptosis **(Figure 5B and 5D)**. However, there are still few studies in this research field, and in-depth studies are needed to elucidate the relationship between protein methylation and ferroptosis and to develop strategies for targeting protein methylation to modulate ferroptosis and treat disease. Moreover, to date, there are few reports on the correlation between non-histone methylation and ferroptosis. For example, although the relationship between P53 and ferroptosis has been well established^88^, ^211^, ^212^, there is no evidence suggesting that its methylation is involved in the regulatory effect of P53 on ferroptosis.

## Conclusions and perspectives

In the present review, we summarize the basis of ferroptosis and the evidence acquired to date to show how DNA, RNA, and protein methylation regulate ferroptosis. It is clear that methylation modification of all three types of molecules is essential for the regulation of ferroptosis and that these modified molecules exert influence mainly by regulating the expression of ferroptosis-associated proteins. There are at least six key unanswered scientific questions for which the answers are likely to drive advances in understanding the epigenetic regulation of ferroptosis in the near future. First, how do DNA, RNA and protein methylation mutually regulate and synergistically influence ferroptosis under certain conditions, and what is the spatiotemporal regulatory relationship among them? Second, what are the mechanisms by which DNA methylation in different regions during ferroptosis regulates related gene expression? Specifically, how methylated DNA in coding regions affects the expression of these genes should be investigated further. Third, what determines the specificity of the RNA m^6^A modification sites in ferroptosis, and how do RNA m^6^A modification sites and reader proteins jointly regulate the fate of related mRNAs in this context? Fourth, are methylated non-histone proteins, in particular those non-histones that have been linked to ferroptosis, involved in the regulation of ferroptosis? Fifth, how do other epigenetic modifications form a regulatory network with DNA, RNA, and protein methylation to regulate ferroptosis? Sixth, further exploration is needed to determine the potential of targeting DNA, RNA, or protein methylation to regulate ferroptosis and thereby treat and prevent disease. Moreover, whether combined inhibitors/inducers of ferroptosis and inhibitors/agonists of biomolecular methylation can function synergistically to mitigate related diseases needs to be explored in depth. Answers to these questions will not only advance our understanding of ferroptosis but will also provide important new insights into the biological functions related to DNA, RNA, and protein methylation.

## Abbreviations

5mC: 5-methyl-cytosine
5hmC: Hydroxymethyl-cytosine
5fC: Formyl-cytosine
5caC: Carboxyl-cytosine
ACSL4: Acyl-CoA synthetase long-chain family member 4
ALOX12: Arachidonate 12-lipoxygenase, 12S type
ALOX15: Arachidonic acid 15-lipoxygenase-1
ATG3: Autophagy related gene 3
AMPK: AMP-activated protein kinase
AIFM2: Apoptosis inducing factor mitochondria-associated 2
AA: Arachidonic acid
AdA: Adrenic acid
AURKA: Aurora kinase A
ALKBH5: AlkB homolog 5
ATF4: Activating transcription factor 4
BH_4_: Tetrahydrobiopterin
BAPN: β-aminopropionitrile monofumarate
BRD4: Bromodomain containing 4
CDDO: 2-amino-5-chloro-N, 3-dimethylbenzamide
CpG: Cytosine-phosphate-guanine
CM: Cutaneous melanoma
CYBRD1: Cytochrome B reductase 1
COPD: Chronic obstructive pulmonary disease
CSE: Cigarette smoke extract
CBS: Cystathionine β-synthase
DHODH: Dihydroorotate dehydrogenase
DFO: Deferoxamine
DMT1: Divalent metal transporter 1
DNMT3A: DNA Methyltransferase 3A
ER: Endoplasmic reticulum
ERFE: Erythroferrone
EGLN2: Egl-9 family hypoxia inducible factor 2
ELOVL5: Elongation of very long chain fatty acid protein 5
ELAVL1: Embryonic lethal-abnormal vision like protein 1
EMT: Epithelial-mesenchymal transition
EZH2: Enhancer of zeste homolog 2
FPN: Ferroportin
FRGs: Ferroptosis regulator genes
FPI: Ferroptosis potential index
FADS2: Fatty acid desaturase 2
FTO: Fat mass and obesity-associated protein
FTH1: Ferritin heavy chain 1
FSP1: Ferroptosis-suppressor-protein 1
Fer-1: Ferrostatin-1
GPX4: Glutathione peroxidase 4
GSH: Glutathione
GCH1: GTP cyclohydrolase 1
GCLC: Glutamatecysteine ligase catalytic subunit
GBM: Glioblastoma multiforme
GLUT1: Glucose transporter type 1
GCs: Gastric cancer cells
HHcy: Hyperhomocysteinemia
HSCs: Hematopoietic stem cells
HO-1: Heme oxygenase-1
HERC2: HECT and RLD domain containing E3 ubiquitin protein ligase 2
HSP90: Heat shock protein 90
HSPA5: Heat shock protein family A member 5
HNSCC: Head and neck squamous cell carcinoma
HIF-1α: Hypoxia inducible factor-1α
HBE: Human bronchial epithelial
HNRNPA2B1: Heterogeneous nuclear ribonucleoprotein family protein A2/B1
HASMCs: Human aortic smooth muscle cells
HCC: Hepatocellular carcinoma
HOXA13: Homeobox A13
HBx: Hepatitis B virus X protein
I/R: Ischemia/reperfusion
IGF2BP1: IGF2 mRNA-binding protein 1
IGF2BP3: Insulin-like growth factor 2 mRNA binding protein 3
KDM3B: Lysine demethylase 3B
LPCAT3: Lysophosphatidylcholine acyltransferase 3
LOXs: Lipoxygenases
LC3 II: Microtubule-associated protein 1 light chain 3 II
LAMP2a: Lysosome-associated membrane protein 2a
LUSC: Lung squamous cell carcinoma
LSH: Lymphoid-specific helicase
LPS: Lipopolysaccharide
LUAD: Lung adenocarcinoma
MYSM1: Myb like, SWIRM and MPN Domains 1
MUFA: Monounsaturated fatty acyl
MUC1-C: Mucin 1 C-terminal subunit
m^5^C: 5-methylcytosine
m^1^A: N^1^-methyladenosine
m^3^C: N^3^-methylcytosine
m^7^G: N^7^-methylguanosine
METTL3: Methyltransferase-like 3
m^6^A: N^6^-Methyladenosine
MLL: Menin-mixed-lineage leukemia
NRF2: NFE2 like BZIP transcription factor 2
NCOA4: Nuclear receptor coactivator 4
NOXs: NADPH oxidases
NOS: Nitric oxide synthases
NF2: Neurofibromin 2
NKAP: NF-κB activating protein
NETs: Neutrophil extracellular traps
NSCLC: Non-small cell lung carcinoma
NOX2: NADPH oxidase 2
OTUB1: OTU domain-containing ubiquitin aldehyde-binding protein 1
PLOOHs: Phospholipid hydroperoxides
PUFA: Polyunsaturated fatty acid
PRX: Peroxiredoxin
PE: Phosphatidylethanolamine
PINK1: PTEN Induced Kinase 1
PKMTs: Protein lysine methyltransferases
PRMTs: Protein arginine methyltransferases
PA: Palmitic acid
PRMT1: Protein arginine methyltransferase 1
ROS: Reactive oxygen species
RFK: Riboflavin kinase
RAP1A: Ras-related protein Rap-1A
SCD1: Stearoyl-CoA desaturase 1
SLC39A14: Solute carrier family 39 member 14
SQSTM1: Sequestosome 1
SLC7A11: Solute carrier family 7 member 11
SCNA: Somatic copy number alterations
SCARA5: Scavenger receptor class A member 5
SLC11A1: Solute carrier family 11 member 1
SCD1: Stearoyl-CoA desaturase 1
SFPQ: Splicing factor proline and glutamine-rich
SI-ALI: Sepsis-associated acute lung injury
SIRT1: Histone deacetylase Sirtuin 1
SETDB1: SET domain bifurcated 1
STC2: Stanniocalcin 2
Tf: Transferrin
TRIM7: Tripartite motif-containing protein 7
TRX: Thioredoxin
TET: Ten-eleven translocation
TFR2: Transferrin receptor 2
TAAD: Stanford type A aortic dissection
TNF-α: Tumor necrosis factor-α
UGC: Upper gastrointestinal adenocarcinoma
VK: Vitamin K
WTAP: Wilms' tumor 1-associated protein
xCT: System xc^-^
YAP: Yes1 associated transcriptional regulator
YTHDF1: YT521-B homology (YTH) domain family protein F1
